# A landscape genetic analysis of important agricultural pest species in Tunisia: The whitefly *Bemisia tabaci*

**DOI:** 10.1371/journal.pone.0185724

**Published:** 2017-10-03

**Authors:** Ahmed Ben Abdelkrim, Tarek Hattab, Hatem Fakhfakh, Mohamed Sadok Belkadhi, Faten Gorsane

**Affiliations:** 1 Laboratoire de Génétique Moléculaire, Immunologie et Biotechnologie. Faculté des Sciences de Tunis, Université Tunis El Manar, Tunis, Tunisie; 2 Institut Jacques Monod, CNRS UMR 7592, Université Paris Diderot, Sorbonne Paris Cité, Paris, France; 3 Institut Français de Recherche pour l’Exploitation de la Mer, IFREMER, UMR 248 MARBEC, Avenue Jean Monnet CS, Sète, France; 4 Faculté des Sciences de Bizerte, Zarzouna, Université de Carthage, Bizerte, Tunisie; 5 Centre Technique des cultures protégées et géothermiques de Gabes, Gabes, Tunisie; Northwest A&F University, CHINA

## Abstract

Combining landscape ecology and genetics provides an excellent framework to appreciate pest population dynamics and dispersal. The genetic architectures of many species are always shaped by environmental constraints. Because little is known about the ecological and genetic traits of Tunisian whitefly populations, the main objective of this work is to highlight patterns of biodiversity, genetic structure and migration routes of this pest. We used nuclear microsatellite loci to analyze *B*. *tabaci* populations collected from various agricultural areas across the country and we determine their biotype status. Molecular data were subsequently interpreted in an ecological context supplied from a species distribution model to infer habitat suitability and hereafter the potential connection paths between sampling localities. An analysis of landscape resistance to *B*. *tabaci* genetic flow was thus applied to take into account habitat suitability, genetic relatedness and functional connectivity of habitats within a varied landscape matrix. We shed light on the occurrence of three geographically delineated genetic groups with high levels of genetic differentiation within each of them. Potential migration corridors of this pest were then established providing significant advances toward the understanding of genetic features and the dynamic dispersal of this pest. This study supports the hypothesis of a long-distance dispersal of *B*. *tabaci* followed by infrequent long-term isolations. The Inference of population sources and colonization routes is critical for the design and implementation of accurate management strategies against this pest.

## Introduction

The sweet potato whitefly, *Bemisia tabaci*, is an economically important agriculture pest consisting of at least 34 cryptic members [1]. It is highly polyphagous and invasive, colonizing more than 1000 different plant species and causing significant losses by feeding and through acting as a vector for more than 300 plant viruses [2,3]. Based on mitochondrial cytochrome oxidase I (COI) DNA retrieved from sequences of worldwide *B*. *tabaci*, 34 genetic groups have been discriminated [4]. They belong to four major clusters: Sub-Saharan Africa; Asia; the New World and the latest cluster that includes North Africa, the Middle East and Asia Minor [1]. Currently, the global status of this pest refers to two distinct species corresponding to Middle East-Asia Minor 1 (MEAM1, formerly biotype B) and Mediterranean (MED, formerly biotype Q) [1,5]. *B*. *tabaci* is now considered as a complex of well-defined groups that are referred to biotypes and which are distinguishable according to host specialization, reproductive compatibility, differential resistance to different classes of insecticide and efficiency in transmitting phytoviruses [4,6].

Both direct observation and model predictions show that *B*. *tabaci* is expanding its distribution in the Mediterranean basin [7]. Temperature is the major environmental variable that influences the development, survival and reproduction of *B*. *tabaci* populations, and so their potential geographic range [7,8]. Host plant availability and therefore agricultural and land use practices, are also important factors that modulate the population distributions and structure of *B*. *tabaci* across the landscape [9,10]. Understanding how climate and landscape heterogeneity shape the distribution of pest populations and gene flow between landscape patches is crucial in pest management science [11,12]. Answer to this question requires the use of the landscape genetics approach [13–15], which combines concepts and tools from population genetics, landscape ecology, geography and spatial statistics in order to assess how landscape resistance modulates animal species move across the environment [12]. Thus, landscape resistance or its inverse landscape permeability can be modeled to (i) identify landscape and environmental features that constrain genetic connectivity, (ii) and to perform a spatially explicit exploration of genetic diversity and population structure, mainly to inform resource management and conservation [12,16,17].

The population genetics of many pest species are shaped by environmental gradients [18,19]. A combination of genetic diversity information based on molecular markers and environmental approaches therefore has the potential to provide a powerful framework for the study *B*. *tabaci* population dynamics and dispersal in Tunisia. Previously, it was reported that the high divergence between the mitochondrial cytochrom oxidase I (mtCOI) sequences among *B*. *tabaci* clades suggested that ecological factors, particularly climatic and tectonic events, have contributed to the extreme diversification of *B*. *tabaci* [4,6,20]. In Tunisia, the identification and biotype distribution of naturally occurring *B*. *tabaci* population were assessed a few years ago [21]. In depth, analysis of the genetic affiliation of these populations by PCR-RFLP (*Taq*I) of the mtCOI gene and sequencing indicated that the sampled whiteflies clustered into the B and Q biotypes. Genetic investigation driven by several approaches including molecular variance (AMOVA), selective neutrality and genetic haplotype network tests supported the hypothesis that Tunisian *B*. *tabaci* populations underwent a potential expansion followed by gene flow restriction [21].

Although mtCOI markers have been extensively used to provide insights into *B*. *tabaci* groups and thus, could be used as ‘‘integrated barcodes”, they do not answer the same questions as integrated nuclear, morphological, and ecological data sets [22,23]. Such data sets could be useful for confirming hypotheses based on other sources of data. mtDNA markers also have limitation such as maternal inheritance, recombination, inconsistent mutation rates, heteroplasmy and compounding evolutionary processes [22,24]. In contrast, nuclear molecular markers such as simple sequence repeats (SSRs) can be considered as more valuable tools for assessing the genetic structure and traits of natural *B*. *tabaci* populations. Out of the 50 described loci [9,25–29], a panel of seven microsatellite loci, widely used in *B*. *tabaci* genetic studies [28], were selected in this work and scored in naturally occurring populations located in different geographical parts of Tunisia. Results gave further insights into population structure and differential patterns of genetic diversity across defined geographical groups.

Molecular data are also important and useful in conjunction with other sources of information such as ecology data [22,30]. By merging SSR molecular markers with the biotype status of *B*. *tacaci* population and species distribution modeling (SDM) [31], we were also able to analyze potential migration corridors among *B*. *tabaci* populations in Tunisia. We used large-scale SDM to infer the climatic suitability of the landscape for *B*. *tabaci* and pathways based on least-cost distance between individuals with a high level of genetic relatedness. Using this approach, we mapped landscape resistance to *B*. *tabaci* genetic flow and we identified the landscape elements, such as natural barriers, that isolate *B*. *tabaci* populations and the potential connection corridors between sampling localities. These corridors reflected the dual sources of the connection: both genetic distance and environmentally suitable paths of migration inferred from the SDM. Thus, modeling landscape resistance and potential migration corridors represents a major advance for the prioritizing *B*. *tabaci* elimination campaigns, at the national level.

## Materials and methods

### *Bemisia tabaci* samples

**Fig 1:**
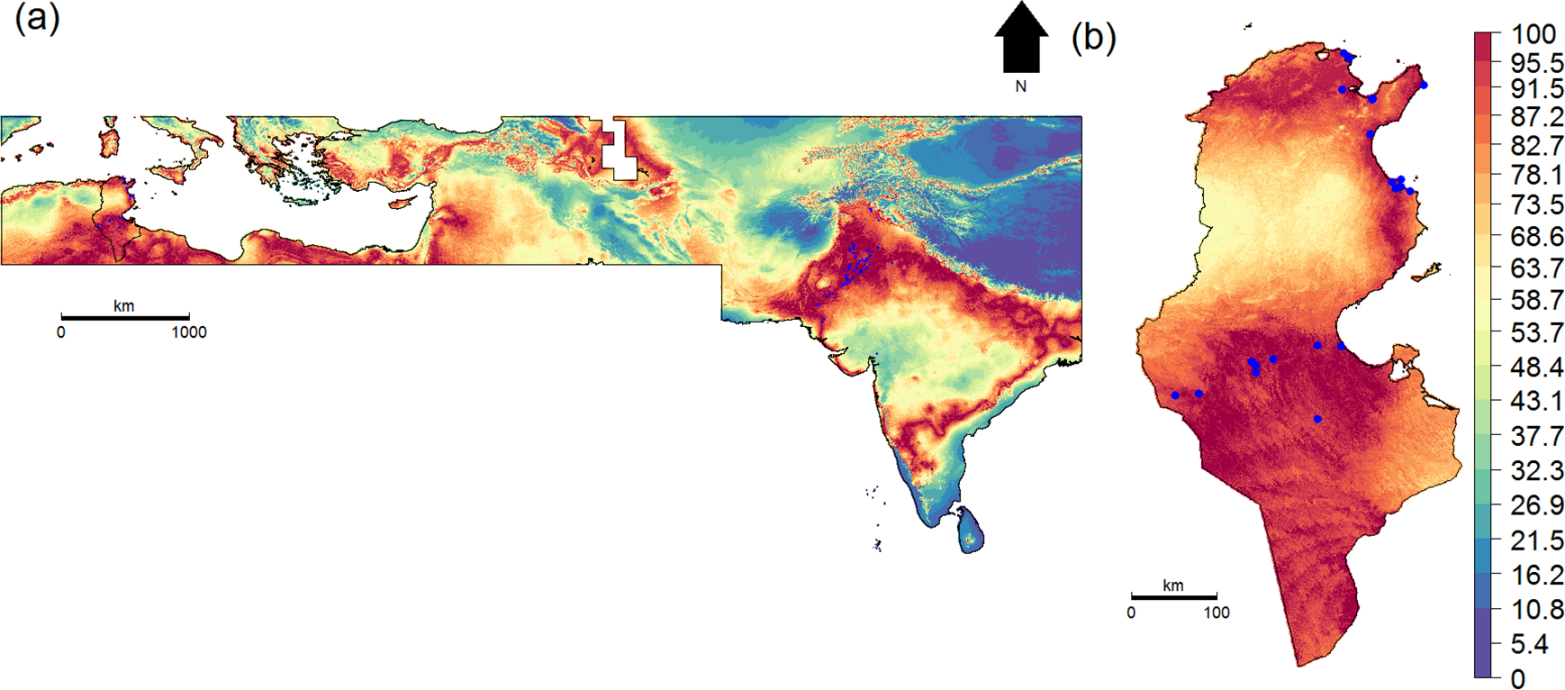
Climatic suitability map of *Bemisia tabaci*. (A) across the geographical extent used for model calibration and (B) across Tunisia. Blue dots represent recorded occurrences.

A total of 1475 female *B*. *tabaci* adults corresponding to 59 distinct populations were collected during 2014–2016 from both open fields and greenhouses (Fig 1). Populations were sampled from 16 localities within the main vegetable-growing areas across Tunisia (Table 1). Whitefly samples were collected alive and immediately preserved in 96% ethanol at 20°C until DNA extraction was performed.

**Table 1:** Collections’ details for the 59 samples of *Bemisia tabaci* from Tunisia.

Sample	Locatity	GPS coordinates	Geographic groups	Host plant	Biotypes
Pop1	Teboulba	35°38'N, 10°56'E	Sahel	Tomato	B
Pop2	Mansoura	36°51'N, 11° 7'E	North	Melon	Q
Pop3	Mansoura	36°51'N, 11° 7'E	North	Cucumber	Q
Pop4	SidiNeji	36°51'N, 11° 7'E	North	Cucumber	Q
Pop5	Manouba	36°48'N, 10° 6'E	North	Melon	Q
Pop6	Bouficha	36°17'N, 10°27'E	North	Tomato	Q
Pop7	Bouficha	36°18'N, 10°27'E	North	Melon	Q
Pop8	RasDjbal	37°12'N, 10° 7'E	North	Zucchini	Q
Pop9	Ghar El Melh	37°10'N, 10°10'E	North	Zucchini	Q
Pop10	Ghar El Melh	37°10'N, 10°10'E	North	Melon	Q
Pop11	Ghar El Melh	37°10'N, 10°11'E	North	Melon	Q
Pop12	El Sahline	35°44'N, 10°43'E	Sahel	Pepper	B
Pop13	Slimane	36°41'N, 10°28'E	North	Cauliflower	B
Pop14	Slimane	36°41'N, 10°28'E	North	Potato	B
Pop15	Slimane	36°41'N, 10°28'E	North	Tomato	B
Pop16	Slimane	36°42'N, 10°29'E	North	Zucchini	B
Pop17	Slimane	36°42'N, 10°28'E	North	Cauliflower	B
Pop18	Monastir	35°40'N, 10°45'E	Sahel	Tomato	B
Pop19	Monastir	35°46'N, 10°49'E	Sahel	Zucchini	B
Pop20	Kébili	33°42'N, 8°57'E	South	Lantana	Q
Pop21	Gabes	33°53'N, 9°47'E	South	Tomato	B
Pop22	Rjim-Maatoug	33°19'N, 8° 0'E	South	Black Nightshade	Q
Pop23	Gabes	33°53'N, 9°47'E	South	Zucchini	B
Pop24	Kébili	33°42'N, 8°57'E	South	Tomato	Q
Pop25	Teboulba	35°38'N, 10°56'E	Sahel	Tomato	B
Pop26	SidiNeji	36°51'N, 11° 7'E	North	Cucumber	B
Pop27	Bouficha	36°17'N, 10°27'E	North	Melon	Q
Pop28	RasDjbal	37°12'N, 10° 7'E	North	Zucchini	Q
Pop30	El Sahline	35°44'N, 10°43'E	Sahel	Pepper	B
Pop31	Slimane	36°41'N, 10°28'E	North	Cauliflower	B
Pop32	Slimane	36°41'N, 10°28'E	North	Potato	B
Pop33	Slimane	36°41'N, 10°28'E	North	Zucchini	B
Pop34	Slimane	36°41'N, 10°28'E	North	Cauliflower	B
Pop35	Monastir	35°46'N, 10°49'E	Sahel	Tomato	B
Pop36	Monastir	35°46'N, 10°49'E	Sahel	Zucchini	B
Pop37	Kébili	33°42'N, 8°57'E	South	Lantana	Q
Pop38	Gabes	33°53'N, 9°47'E	South	Tomato	B
Pop39	Rjim-Maatoug	33°19'N, 8° 0'E	South	Black Nightshade	Q
Pop40	Gabes	33°53'N, 9°47'E	South	Zucchini	B
Pop41	Kébili	33°42'N, 8°57'E	South	Tomato	Q
Pop42	Teboulba	35°38'N, 10°56'E	Sahel	Tomato	B
Pop43	SidiNeji	36°51'N, 11° 7'E	North	Cucumber	B
Pop44	Bouficha	36°17'N, 10°27'E	North	Melon	Q
Pop45	RasDjbal	37°12'N, 10° 7'E	North	Zucchini	Q
Pop46	Ghar El Melh	37°10'N, 10°11'E	North	Zucchini	Q
Pop47	El Sahline	35°44'N, 10°43'E	Sahel	Pepper	B
Pop48	Slimane	36°41'N, 10°28'E	North	Cauliflower	B
Pop49	Slimane	36°41'N, 10°28'E	North	Potato	B
Pop50	Mansoura	36°51'N, 11° 7'E	North	Melon	B
Pop51	Kébili	33°20'N, 8°18'E	South	Melon	Q
Pop52	Kébili	33°34'N, 9° 1'E	South	Melon	Q
Pop53	Kébili	33°42'N, 8°58'E	South	Lantana	Q
Pop54	Gabes	33°52'N, 10° 5'E	South	Zucchini	B
Pop55	Kébili	33°44'N, 9°14'E	South	Melon	Q
Pop56	Kébili	33°44'N, 9°14'E	South	Tomato	Q
Pop57	Kébili	33°39'N, 9° 0'E	South	Melon	Q
Pop58	Gabes	33°53'N, 9°47'E	South	Tomato	B
Pop59	Gabes	33°52'N, 10° 5'E	South	Tomato	B
Pop60	Kébili	33°42'N, 8°58'E	South	Melon	Q

Pop: population

**Solanaceae**: Tomato (*Solanum lycopersicum*.), Eggplant (*Solanum melongena*), pepper (*Capsicum annuum*), Potato (*Solanum tuberosum*), Black Nightshade (*Solanum nigrum*); **Cucurbitacea**: Melon (*Cucumismelo*), Zucchini (*Cucurbita pepo*), **Brassicacea**: Cauliflower (*Brassica oleracea*) and **Verbenacea** (*Lantana camara*).

### DNA extraction

Twenty-five adult insects corresponding to one population were frozen in liquid nitrogen before homogenization in an extraction buffer (100 g/l Proteinase K; 0.45% Triton; 0.45% Tween; 1M Tris-HCl, pH: 8). The mixture was incubated for 60 min at 55°C, then at 100°C for 10 min, and finally at 0°C for 5 min. A centrifugation step was performed at 1,000 rpm for 5 min to pellet debris. DNA quantification was performed with ND-1000 spectrophotometer (Nanodrop Technologies, USA).

### Identification of *B*. *tabaci* biotypes

The Biotype status of *B*. *tabaci* samples was determined through (i) The PCR- RFLP (*Taq*I) of a fragment of the mitochondrial gene (mtCOI) [32] and (ii) The PCR amplification of a microsatellite fragment BEM23- (GAA)_31_ imp [33]. For the first method, PCR reaction was performed in a final volume of 25 μl containing 100 ng of each primer [34], 5 μl of 10X buffer, 1 μl of dNTP (10 mM each), 1.5 μl MgCl2 (1.5 mM), 1Uof *Taq* DNA polymerase (MP BIOMEDICALS), 2 μl of extracted DNA, and sterile water. PCR amplifications were performed on a T professional trio system thermalcycler (Biometra, Germany) using the following cycling program: One first denaturation step of 4 min at 94°C followed by 35 amplification cycles (30 s at 94°C, 50 s at 55°C, 1 min at 72°C). A final step of extension is performed at 72°C for 10 min. PCR products were purified (PureLink PCR Purification Kit, Invitrogen, Paris, France) and digested with 1 U of *Taq*I restriction enzyme with 2 μl of the corresponding buffer (10X) in a final volume of 20 μl, for 2 h at 65°C. PCR products or cleaved fragments were separated in 2% agarose gel and visualized by staining with ethidium bromide. For the second approach, microsatellite marker amplifications were carried out using primers developed by [33] and validated by [35, 36]. PCR-amplified alleles were scored as described below.

### Simple Sequence Repeat (SSR) genotyping

Seven microsatellite markers were selected based on their previous use [9]. Amplifications were carried out using the PCR conditions described by [28]. A green microfluidic chip was used following the DNA 1K analysis kit protocol. For each microsatellite marker, PCR-amplified bands were identified and scored by the Experion System (Automated Electrophoresis Station). Electrophoregrams were analyzed using the Experion software, System Operation and Data Analysis Tools (version 1.0).

### Genetic analysis and population structure

To evaluate the genetic diversity of *B*. *tabaci* populations, the basic genetic population parameters such as the number of alleles detected per locus (Na), the polymorphism information content (PIC) values, the observed (Ho) and the expected heterozygosity (He) as well as significance values for deviations from the Hardy–Weinberg equilibrium (HWE) were determined.

Multiple co-inertia analysis (MCOA) approach was performed to identify alleles characterizing geographic groups and to evaluate the efficiency of each marker to build population structure since this technique allows each locus to be analyzed separately [37]. Then, the proportion of variance attributable to genetic differences between and among the main geographical regions (North, Sahel and South) was estimated, for each biotype, using hierarchical analysis of molecular variance (AMOVA). To cluster sampled populations into genetic groups we used a discriminant analysis of principal components (DAPC). To achieve that, principal component analysis (PCA) was used to transform the raw SSR data into a set of linearly uncorrelated variables. Afterwards, we identified the optimal number of clusters that minimize the variation within clusters using the Bayesian Information Criterion (BIC). A discriminant analysis was then applied to 14 principal components, explaining 59.4% of the total variance of the SSR data. Based on the retained discriminant functions, a membership probability of each population to each cluster was calculated, which can be interpreted in order to assess how clear-cut or admixed the clusters are [38].Visualization of the relationships between population and clusters in the DAPC space were realized using a scatterplot. For the visualization of relationships between genetics groups, we calculated Nei’s genetic distances [39] between groups and we constructed a neighbor-joining tree.

### Species distribution modeling

Ecological niche factor analysis (ENFA) [40] was used to create a climatic suitability map that depicts areas where *B*. *tabaci* is unlikely to occur. ENFA is a specific multivariate ordination technique that relies on presence-only data to compare a species’ environmental niche and the environmental characteristics of the studied area. To this end, ENFA compares the distribution of the environmental predictors between the occurrence locations and the whole studied area and assigns a degree of suitability to each point on a map (typically from 0 to 100). We used as environmental predictors 19 bioclimatic variable data provided by WorldClim database (version 1.4) [41]. This bioclimatic dataset is derived from monthly temperature and rainfall value data for the period 1950–2000 and represents annual trends, seasonality and extreme or limiting environmental factors. In particular, we used bioclimatic surfaces with pixel size of 30 arc seconds corresponding to a resolution of approximately 1 km and covering North Africa, the Middle East and part of Asia (Fig 1). This geographical extent was chosen for two reasons: (1) it matches the range of distribution of one of the high-level genetic groups of *B*. *tabaci* [1] (2) it is advised to develop SDMs across the range of climatic conditions in which a given species occurs [42,43].

To calibrate the ENFA model, we merged our spatial genetic dataset with 207 geographical occurrences located within the bioclimatic surface areas and obtained from the Global Biodiversity Information Facility (GBIF; http://data.gbif.org, 2016) (Fig 1). To evaluate the accuracy of the ENFA, we performed a ten-fold cross validation based on the Boyce index, which measures how far model predictions differ from random distribution of the observed presences across prediction gradients [44]. It is continuous and varies between -1 and +1. Positive values indicate a model whose predictions are consistent with the presence distribution in the evaluation dataset. Values close to zero mean that the model is no different from a chance model, while negative values indicate an incorrect model, which predicts poor quality areas where presences are frequent [45].

### Predicting population connection corridors

Climatic suitability cells located in Tunisia were first taken from the ENFA prediction map. Given the strong link between *B*. *tabaci* and the great number of host species for this phytophagous insect [46], we assigned zero values of habitat suitability to cells located outside vegetation areas in Tunisia. Land cover data were obtained from the GLCN Land Cover datasets (http://www.glcn.org/databases/lc_gc-africa_en.jsp) at 300-m resolution. The resulting map, reflecting both abiotic (i.e. temperature and rainfall) and biotic (host and/or prey availability) constraints, was used to create a resistance surface. To this end, a transition matrix was computed from the habitat suitability values to define the connectedness between adjacent pixels. We used King's graph as a neighborhood function, in which a given pixel is considered to be connected to the eight adjacent pixels. Each cell on a resistance surface is assigned a cost value, with high costs given to unsuitable cells that restrict dispersal and low costs to suitable cells that facilitate dispersal [47]. Thus, higher resistance values represent specific factors that limit the ability of an individual *B*. *tabaci* to traverse a cell, which could be in this case a function of mortality risk, energetic costs, food availability and interactions between each of these factors.

Based on a Wright's F_ST_ paire-wise genetic distance matrix, we extracted likely genetically identical samples: pairs with Wright's F_ST_ index [48] lower than percentile 5% of all pair-wise comparisons. Then, using the resistance surface we calculated accumulated cost surfaces between each pair of these genetically related samples using Dijkstra's algorithm[49,50]. The accumulated cost surfaces are a measure of nearness that optimizes the geographical distance travelled and the costs traversed [50]. The resulting accumulated cost surfaces were then normalized between 0 and 1 to ensure comparability between the different resistance sets. Finally, all corridors were merged in one map representing the network of potential connection corridors between sampled populations.

All data processing and statistical analysis were performed using R 3.1 [51].

## Results

### Genetic analysis

We analyzed 59 *B*. *tabaci* populations collected on different hosts throughout the country and clustered them into three major geographic groups: North, Sahel and South (Table 1). *B*. *tabaci* populations were assigned to either biotype B or Q according to PCR-RFLP (*Taq*I) patterns of the mtCOI gene fragment in conjunction with a diagnostic microsatellite marker. Two restriction patterns (414, 162, 144 bp) and (631,144 bp) corresponding to biotype B and Q respectively, were revealed (S1 Fig). Results were corroborated by Bem23 micosatellite analysis since the alleles > 371 bp were scored only within Q biotype. Biotype B was detected in the three geographic groups, all of which were on vegetables. Biotype Q detection was restricted to the North and the South of the country, on both vegetables and ornamentals (Table 1)

In addition, seven loci were selected to analyse populations’ structure. These gave clear patterns of discrimination between the sampled geographical groups. A total of 113 alleles were scored across the studied populations. The number of alleles (Na) per locus ranged from 6 (detected in locus 177 in the Sahel) to 18 (detected in locus BT4 in the North). The North displayed the highest average Na value (13.42) while the Sahel showed the lowest (8.00). The observed (Ho) and expected (He) heterozygoties ranged from 0.001 to 1.00 and from 0.74 to 0.92 respectively. Regarding polymorphism information content (PIC), all considered loci displayed high values, ranging from 0.82 to 0.92, attesting to the effectiveness of these SSR markers for detecting variability (Table 2).

**Table 2:** Genetic diversity parameters calculated for tested microsatellite loci.

		North	Sahel	South	Overall
**Locus.53**^a^	Na	9.000	7.000	10.000	12.000
	He	0.863	0.835	0.842	0.877
	Ho	0.897	1.000	0.850	0.898
	*P-value*				0.106
	Pic				0.855
**Locus.68**^b^	Na	17.000	9.000	13.000	19.000
	He	0.914	0.860	0.905	0.924
	Ho	1.000	1.000	1.000	1.000
	*P-value*				0.555
	Pic				0.910
**Locus.145**^b^	Na	16.000	7.000	9.000	17.000
	He	0.877	0.785	0.747	0.865
	Ho	1.000	1.000	1.000	1.000
	*P-value*				0.001*
	Pic				0.845
**Locus.177**^b^	Na	10.000	6.000	11.000	11.000
	He	0.777	0.760	0.885	0.866
	Ho	0.552	0.500	0.950	0.678
	*P-value*				0.001*
	Pic				0.844
**Bem23.A1**^c^	Na	10.000	6.000	10.000	14.000
	He	0.843	0.755	0.816	0.849
	Ho	0.379	0.100	0.350	0.322
	*P-value*				0.014*
	Pic				0.825
**BT4**^d^	Na	18.000	11.000	15.000	21.000
	He	0.920	0.885	0.901	0.932
	Ho	0.897	0.900	0.850	0.881
	*P-value*				0.308
	Pic				0.920
**BT159**^d^	Na	14.000	10.000	16.000	19.000
	He	0.870	0.885	0.914	0.912
	Ho	0.793	0.800	0.750	0.700
	*P-value*				0.217
	Pic				0.897
**Mean NA**		13.429	8.000	12.000	
**Mean HE**		0.866	0.824	0.859	
**Mean HO**		0.788	0.757	0.821	

NA: Number of alleles detected; HE: Expected heterozygosity; HO: Observed heterozygosity

*: significant P values; Non-significant P values are not indicated. PIC: Polymorphic index content.

^a^[26]

^b^[28]

^c^[33]

^d^ [25].

Out of the 113 revealed alleles, 19, 57 and 29were not detected in the North, the Sahel and the South, respectively. Subsequently, we outlined the most group-specific alleles for each region corresponding to 23 alleles in the North, 11 alleles in the South and only 3 specific alleles in the Sahel (Table 3).

**Table 3:** Alleles inferred from loci that were specifically revealed within each geographic group.

	Locus 53	Locus 68	Locus 145	Locus 177	Bem23	BT4	BT159
**North**	132	185-193-209-216-233-246	167-176-179-210-223-237-243		205-271-386	334-356-362-365-368	272–291
**Sahel**					202	330	251
**South**	167–170	230			207-371-379	304	211-218-255-274

### Genetic relationships and population structure analysis

**Fig 2:**
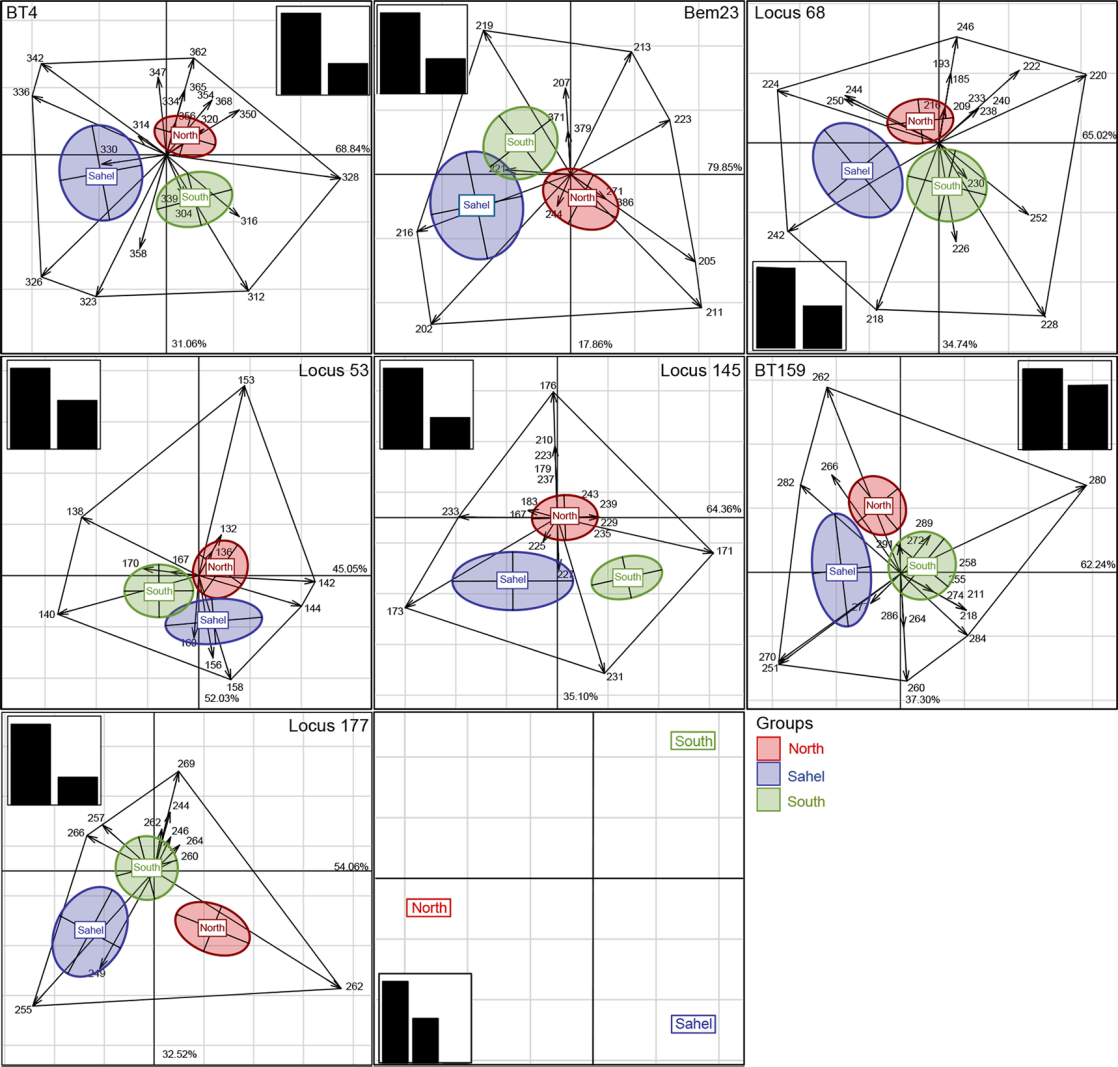
Multiple co-inertia analysis (MCOA) analysis. Single markers coordinated for the first two axes of the PCA. Corresponding plots are drawn on the same scale for the seven markers involved. The first two axes of the % PCA are shown. The three groups of populations are labeled within confidence ellipses (P = 0.95), with an envelope formed by the most discriminating alleles that are joined by lines. The shown barplot of Eigen-values indicates the relative magnitude of each axis with respect to total variance. Distribution of groups based on the common congruence values (Cos^2^) for the two components of the first axis.

We next addressed the capacity and efficiency of marker panels to exhibit genetic structure and to assess the contribution of each specific marker by MCOA according to [37]. This approach permits the identification of alleles of interest that contribute most to the genetic topology of *B*. *tabaci* populations, based on allelic frequencies and single-marker analysis (Fig 2). For example, with regard to the BT4 locus, the first axis accounts for 68.84% of total variance while the second axis accounts for 31.06%. The three geographic groups seem to be mainly structured by seven alleles corresponding to 312, 323, 326, 328, 336, 342 and 362. Regarding the locus Bem23, the percentages of variance derived from the two first axes were 97% and the three geographic groups were mainly structured by seven alleles (219, 213, 223, 211, 205, 211, 202 and 216). Each SSR locus was interpreted separately allowing us to spotlight alleles that contributed the most to discriminating the three geographic groups. Among revealed alleles, several are located close to the center of the MCOA two-dimensional space and are considered as rare and group-specific alleles (Fig 2).

Based on the three geographic groups of *B*. *tabaci* populations, the AMOVA analysis was performed for each of the two invasive biotypes separately. When considering the B and Q biotypes, the largest part of variation was clearly observed within populations (88.241% and 89.956%, respectively) whereas variation among localities within geographic groups accounted for 0.141% and 2.063%, respectively. Results also showed that genetic differentiation among the three geographic groups was very limited (Table 4).

**Table 4:** Analysis of molecular variance (AMOVA) for biotype B and biotype Q populations of *B*. *tabaci*.

	Source of variation	Sum of squares	Variance components	Percentage variation
**Biotype B**	Among geographical groups	7.577	0.018	0.576%
	Among localities within groups	13.663	0.004	0.141%
	Among populations within localities	81.308	0.339	11.042%
	Within populations	84.000	2.710	88.241%
	Total	186.548	3.071	100.000%
**Biotype Q**	Among geographical groups	6.542	0.084	2.647%
	Among localities within groups	11.289	0.065	2.063%
	Among populations within localities	73.508	0.169	5.334%
	Within populations	80.000	2.857	89.956%
	Total	171.339	3.176	100.000%

**Fig 3:**
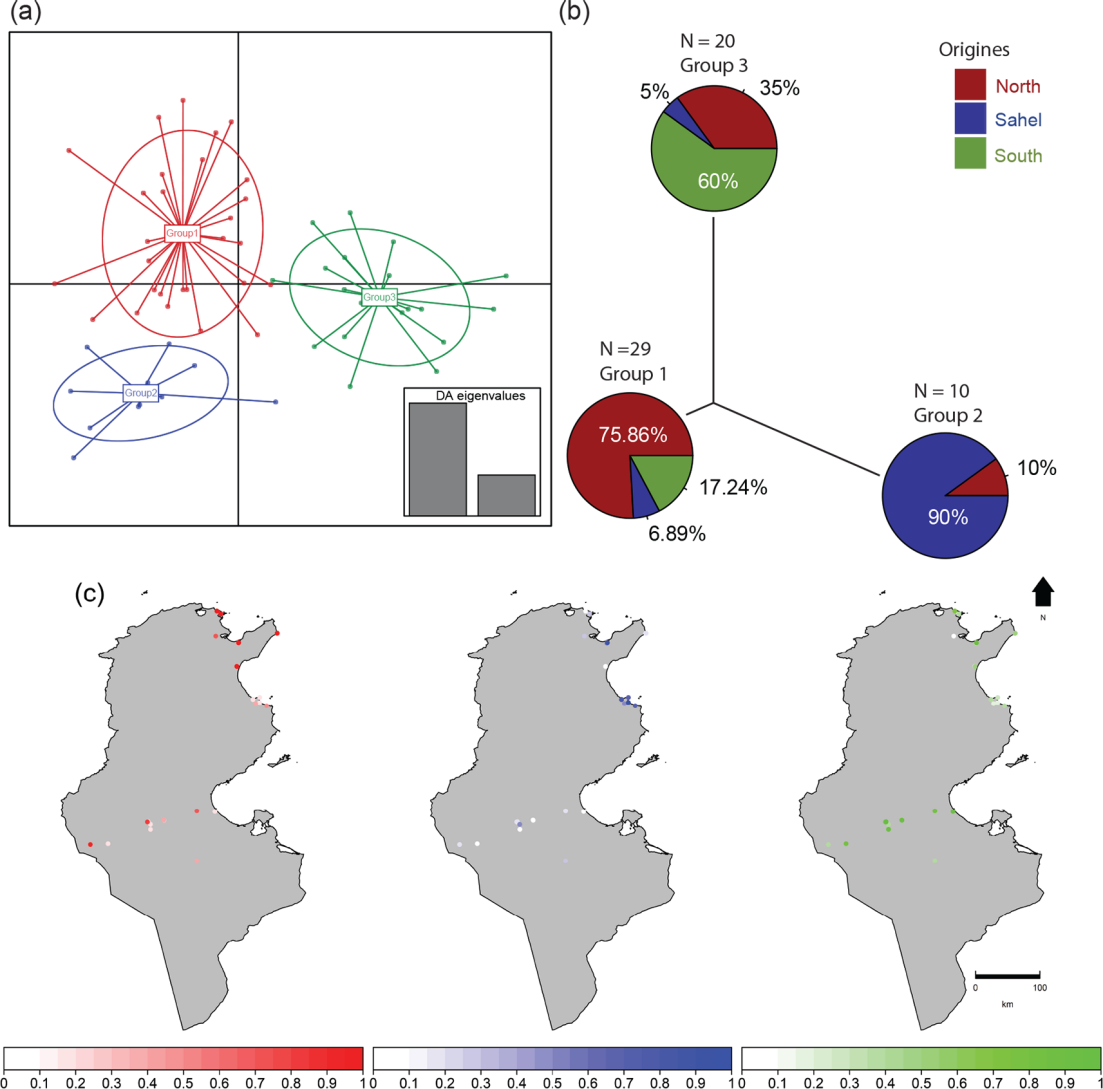
Discriminated analysis of principal components (DAPC) using SSR-based genotypes. (a) DAPC first and second ordination axes. (b) Neighbor-joining tree constructed on the DAPC distances representing the genetic relationship between the inferred 3 clusters as well as the distribution of geographic groups within each of them. (c) Location of the populations (points) and their probabilities for membership in each geographic groups. The colors correspond to the genetic clusters defined by the DAPC analysis.

DAPC analysis was performed to evaluate the overall pattern of variation among natural populations and thus infer the relationships between them. DAPC results supported the existence of three genetic groups (Fig 3A). DAPC was able to assign more than 60% of all populations where they were sampled into the corresponding group. The first group (group 1) was composed of 76.86% of the North population, 17.24% of the South population and 6.89% of the Sahel population. The second group (group 2) was composed of 90% of the Sahel population and 10% of the North population. The last group was composed of 60% of the South population, 35% of the North population and 5% of the Sahel population (Fig 3B). With regard to the genotypes assigned to each group, the North group presented cross-links with both the Sahel and the South (Fig 3C).

### Species distribution modeling

**Fig 4:**
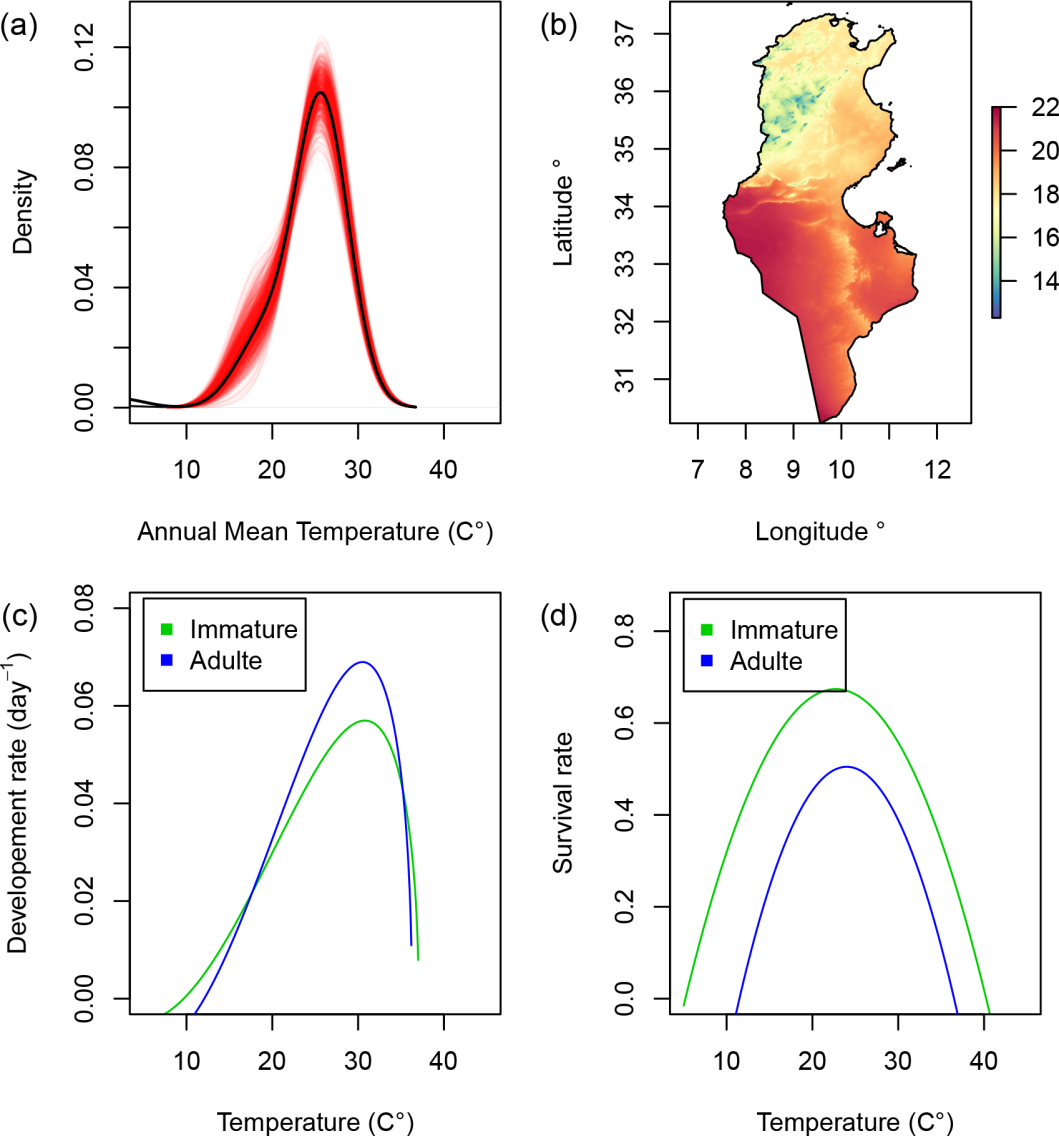
(a) Gaussian kernel density estimates of annual mean temperature values within occurrence data. Red lines represent the result of 1000 permutations based on random samples of the initial occurrence data (70%). (b) Annual mean temperature map. (c) Development rate and (d) survival rate curves as a function of temperature (in°C) for immature and adult whiteflies estimated by [7].

An ENFA analysis was performed and used to create climatic suitability map that depicts broad-scale climatically suitable areas for *B*. *tabaci* (Fig 1). The ten-fold cross validation procedure indicated a good ENFA model quality according to the calculated Boyce index mean and standard deviations of 0.77 ± 0.014. These high values imply that the habitat occupied by *B*. *tabaci* clearly differ from the average environmental conditions found in the broader study area. This indicates that a species-specific habitat selection process takes place. The occurrences records used to calibrate the ENFA model indicate that *B*. *tabaci* is limited by an annual mean temperature of 10–36°C (Fig 4A). The broad-scale distribution shows that high latitude regions, due to their low temperature, can be considered as beyond the climatic limits of the pest. These include the eastern and western Ghats and Himalayan Mountains in India, the Zagros Mountains in Iran, Iraq and south-eastern Turkey and the Atlas Mountains in North Africa (Fig 1A). In Tunisia, climatically suitable areas display a discontinuity in the West-Central region. This is likely due to the lower temperatures of the region, which is an extension of the Atlas Mountains (Figs 1B and 4B).

### Identification of dispersal paths

**Fig 5:**
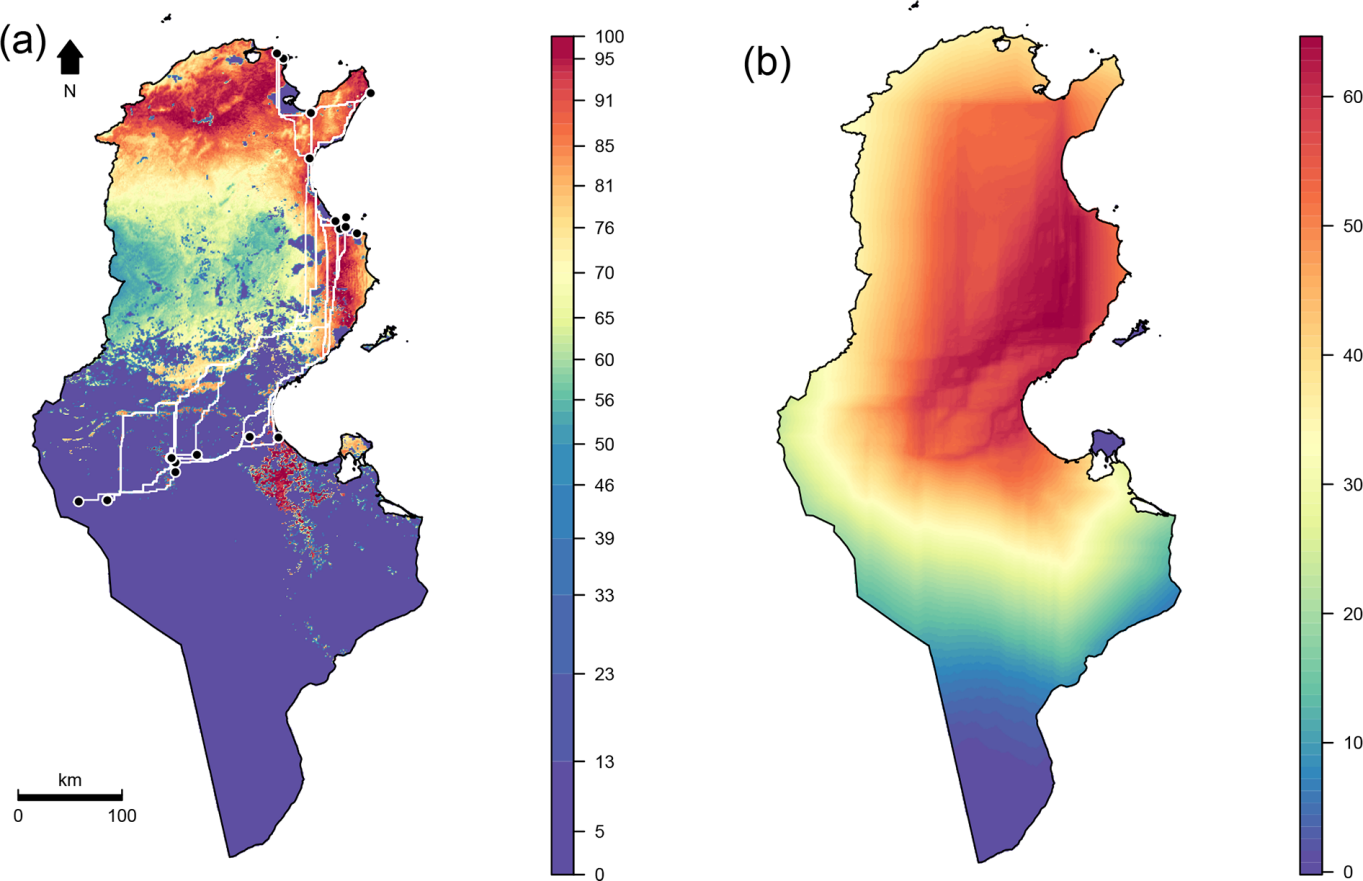
(a) Habitat suitability map of *Bemisia tabaci* reflecting climatic and host/prey constraints. The color gradient represent an habitat suitability index (high values indicate high habitat suitability). White lines represent the shortest path between each pair of genetically related samples. (b) Map of potential migration corridors between sampled populations. The color gradient represent a connectivity index (high values facilitate gene flow and low values indicate low populations connectivity).

A maximum F_ST_ value of 0.27 was set as thresholds to select genetically related samples used for corridors’ construction, thus pairs of localities with pair-wise F_ST_> 0.27 were filtered out. Accordingly, a total of 77 pair-locations from the 1711 possible pair-wise combinations were retained to calculate accumulated cost surfaces (Fig 5A). These results outlined potential *B*. *tabaci* dispersal corridors in Tunisia (Fig 5B). Regions located in the North displayed strong connectivity with the South and Sahel geographic groups as 65 out of 77 least-cost corridors pass through these regions. The gene flow between the northern and southern populations is therefore predicted to take place through indirect paths crossing the Sahel region and to a lesser extent through direct paths crossing the central part of the country (Fig 5B).

## Discussion

Analysis of the genetic structure of a large representative sample of *B*. *tabaci* populations throughout the agricultural areas of Tunisia was carried out to generate new insights into the traits and population dynamics of this pest.

The typical short-generation time of insects makes their population dynamics highly sensitive to climate variability and landscape configuration [52]. In this work, we developed a genetic landscape analysis of *B*. *tabaci* populations whose purpose was to predict the potential migration corridors between populations throughout Tunisia. By using SSR molecular markers and geographical data, our approach combined the analysis of genetic population diversity and structure, reflecting the amount of genetic exchanges between different *B*. *tabaci* populations and SDM predictions. The effect of both temperature rainfall and host/prey availability on the spatial distribution of populations was then considered to take into account the functional connectivity of habitats within a varied landscape matrix to subsequently identify potential connection corridors between groups of *B*. *tabaci* populations.

*B*. *tabaci* was previously described in Tunisia [21,53], but no exhaustive data were available regarding its features. This first investigation of genetic diversity of Tunisian populations of *B*. *tabaci* was performed using PCR-sequencing of mitochondrial cytochrom oxidase I (mtCOI). It was assumed that the largest part of variability could be attributed to differences between localities devoted to each geographic group rather than between groups themselves [21,53]. Beyond cytoplasmic DNA markers, the present work is also based on nuclear SSR markers which are more suitable and accurate for the study of the genetic structure of whitefly *B*. *tabaci* populations [54]. SSR markers are often used for the genetic analysis of insect populations and are effective at differentiating populations [55]. Indeed, SSR markers have been useful in revealing the distribution patterns and genetic structure of *B*. *tabaci* populations on the island of La Réunion [26], around the Mediterranean basin [56, 57,58] and have also enabled the analysis of the variability of these markers to infer the geographical structure within the Asia–Pacific genetic groups [9].

Based on allelic frequencies of single-markers, the MCOA approach allowed the identification of those alleles that contribute most to the genetic topology of *B*. *tabaci* populations. The MCOA two-dimensional space is a useful graphical tool that compares the usefulness of each marker for discriminating populations. This method provides a systemic view of the whitefly population structure focusing on the part of each allele in building it. Table 3 and the Fig 2 show that the frequency of 4 specific alleles allowed the separation of the population from the North (allele 362 of the locus BT4, allele 205 of the locus Bem23, allele 246 of locus 68 and allele 176 of locus 145). Besides, only the alleles 251 of locus BT159 and 202 of locus Bem23 allowed the separation of the Sahel population from the other two populations. Considering each SSR marker separately, the MCOA analysis showed that group-specific alleles are located close to the centre of the plot. Indeed, the Bem23 alleles (386, 379 and 371) that are specific to the Q biotype are placed in the centre of the North and the South populations across the MCOA ordination space. In spite of this, the exhibited structure seems to be more devoted to the efficiency of common alleles to cluster populations into three distinct groups. When all the SSR markers are taken into account, their usefulness to fit in a common and similar structure is achieved even if their efficiency differs. Out of the seven SSR markers, loci 145 and 177 are good structuring markers that displayed three well-defined clusters with a minimum of five discriminating alleles each (176/233/173/171/231 and 257/266/269/262/255, respectively). Overall, MCOA approach is a valuable tool for the selection of efficient markers and the removal of those which are less informative [37].

Our results also suggested that there was a low level of geographic isolation between the geographic groups. The major part of genetic diversity was observed at the population level within geographical groups for both the B (88.24% of the total variance) and the Q (89.96% of the total variance) biotypes. Only 60% of defined genotypes were assigned to the geographic group where they were collected. Genotypes therefore displayed variability within the same geographic group and could be closely related to genotypes in distinct geographic groups.

DAPC results show that Tunisian populations are split into three well-differentiated genetic groups with low overlap between them. Patterns of population structure inferred from nuclear SSRs are in line with the biotype distributions derived from mitochondrial mt COI analysis. Biotype B occurs within the three delineated geographical groups whereas Biotype Q is excluded from the Sahel and seems to be restricted to the north and the south. Indeed, the first genetic group obtained by the DAPC was composed of 51.72% of biotype Q and 48.28% of biotype B, the third group was composed of 65% of biotype Q and 35% of biotype B whereas the second group was composed only of populations of biotype B. Each genetic group seems to possess several specific alleles that may indicate long-distance dispersal followed by infrequent long-term isolation [59,60]. The predicted climatic suitability map of *B*. *tabaci* using the ENFA model showed geographical patterns of distribution similar to those obtained by [7] in the Northern Mediterranean using a temperature-dependent physiologically-based demographic model. Although in this study we developed a correlative SDM that statistically linked environmental data to species distribution records, its predictions fit well with those derived from the mechanistic model described by [7] that integrated the effect of temperature on physiological and demographic processes. By using only spatial observations, the correlative SDM we developed provided a good estimate of the physiological limits of *B*. *tabaci*. The kernel density estimates of annual mean temperature values within occurrence data show thermal tolerance limits between 10–37°C with an optimum at 26°C (Fig 4A). These limits fit well with the biodemographic parameters previously assessed (Fig 4C and 4D) that mechanistically linked *B*. *tabaci* populations to their environments [8]. Indeed, the survival and development rates of *B*. *tabaci* adult stage drop to zero at temperatures values colder than 11° C and warmer than 37° C.

The climatic suitability map predicted by the SDM, after being filtered by vegetation areas (reflecting host and/or prey availability), was used to create a resistance surface (Fig 5A) and then accumulated cost surfaces between each pair of genetically related samples (Fig 5B). This led to the creation of potential migration corridors between localities that take into account habitat suitability, geographic distance, and genetic relatedness. The resulting map of potential migration corridors of *B*. *tabaci*, when interpreted in conjunction with population structure analysis, suggests that *B*. *tabaci* migrates along South-North and Sahel-North potential corridors. The biotype distributions also suggested that the Sahel-North migration corridors concern exclusively the B biotype as the Q one is absent in the Sahel. Otherwise, both B and Q biotypes seem to follow South-North migration corridor. Our results also show that the genetic groups characterizing the southern populations are absent in the Sahel region even though no physical barrier hampers the migration of whiteflies from neighboring areas. The low genetic flow between the Southern and the Sahel regions could be explained by the direction of the prevailing winds in these regions. Small insects are generally thought to be passive fliers and are therefore dependent on air currents to carry them to new sites. *B*. *tabaci* weighs approximately 33 μg and it has been shown that its patterns of migration would be strongly influenced by the wind [61]. This is also in line with our previous work [21]. Our data suggested a possible migration pattern of *B*. *tabaci* from the South to the North regions of the country which was correlated with the spread of *Begomoviruses* vectored by whiteflies. Other studies [9] suggested a South to North trajectory reflecting the direction of prevailing winds during warmer seasons when *B*. *tabaci* is more active. Otherwise, the genetic structure identified in this study may also be explained by plant trade which can act as a homogenizing factor, erasing *B*. *tabaci* population genetic structure between geographic groups [28]. Indeed, the presences of northern populations in the South or the southern population in the North are additional pieces of evidence that human activities may deeply impact the population genetics of *B*. *tabaci* in Tunisia.

The knowledge gained during this study have several applications for pest management. First, by developing a map of the potential distribution of *B*. *tabaci*, we have provided the first pest risk analysis of *B*. *tabaci* to plant health in Tunisia. The predictions of areas suitable for establishment can be used to support pest management tactics and strategies such as incursion monitoring in areas not yet infested but having a high risk of infestation (cf. as is the case for north-western regions). However, it is important to note that predictions made in this study reflect only the potential distribution of B. *tabaci*. Thus, we propose in the future to refine the method by also mapping the current distribution of *B*. *tabaci*. Accurately mapping such distribution and projecting the potential distribution of *B*. *tabaci* are both relevant tasks for managing this plant pests. For instance, control and eradication efforts should focus on the current distribution while containment efforts should focus on the interface between the current and potential distributions [62]. Mapping the current distribution will require a better knowledge of host species and *B*. *tabaci* interactions and the integration of wind effects on dispersion using for instance Lagrangian dispersion models.

The thorough understanding of whitefly population structure and migration corridors of whiteflies is also particularly important for proper management guidance to plan an effective integrated pest management strategy (IPM) in Tunisia. Today, particular attention is paid to enhancing biological control methods. This would require the use of *B*. *tabaci* predators in crops to decrease both pest population densities and dispersal. Indeed, the use of host plants infested by predators early in the season, in the proximity of crops which are often infested by whiteflies and taking into account preferential routes of migration would permit predators populations to be established prior to whitefly attack. To fully exploit an IPM over a long time scales, knowledge of the parasitic or predatory natural enemies of *B*. *tabaci* should be further investigated. Generalists ‘predators have been shown to reduce pest pressure and to enhance natural enemy activities [63]. They act through strong competition for resources reducing pest pressure on crops and preventing new infestations by invasive pests [64]. Employing multiple natural enemies could ensure continued suppression of a target pest throughout its lifecycle [65,66]. Indeed, the effectiveness of mixed releasing of *B*. *tabaci* biological control agents corresponding to a mixture of the polyphagous predatory *Coleoptera* and two whitefly-specific parasitoids have been tested to be implemented in the design of biological pest control plans [67]. Exploring scenarios that include different natural enemy combinations with different migration patterns (South-North and Sahel-North) may greatly help biological control strategies against this damaging and invasive pest species in Tunisia.

## Supporting information

S1 Fig The PCR- RFLP (*Taq*I) of a fragment of the mitochondrial gene (mtCOI). L: Ladder 100 bp, Biomatik. Pattern 1: 634bp, 144bp corresponding to Q biotype. Pattern 2: 440 bp, 220bp, 144 bp corresponding to B biotype. (-): Negative control: mtCOI amplified fragment (778 bp) without *Taq*I digestion.
(TIF)
